# Growth patterns from birth to overweight at age 5‐6 years of children with various backgrounds in socioeconomic status and country of origin: the ABCD study

**DOI:** 10.1111/ijpo.12635

**Published:** 2020-04-01

**Authors:** Tanja G. M. Vrijkotte, Adriëtte J. J. M. Oostvogels, Karien Stronks, Tessa J. Roseboom, Michel H. P. Hof

**Affiliations:** ^1^ Department of Public Health Amsterdam UMC, University of Amsterdam, Amsterdam Public Health Research Institute Amsterdam The Netherlands; ^2^ Department of Clinical Epidemiology Bioinformatics & Biostatistics, Amsterdam UMC, University of Amsterdam, Amsterdam Public Health Research Institute Amsterdam The Netherlands; ^3^ Department of Gynaecology and Obstetrics Amsterdam UMC, University of Amsterdam, Amsterdam Reproduction & Development Research Institute Amsterdam The Netherlands

**Keywords:** children, growth patterns, overweight, social inequalities

## Abstract

**Introduction:**

Children from minority groups are at increased risk of overweight. This study compared BMI growth patterns from birth onwards of boys and girls with overweight at 5‐6 years, according to socioeconomic status (SES) and country of origin, in order to gain more insight into the critical periods of growth to overweight.

**Methods:**

A total of 3714 singletons of the multi‐ethnic ABCD study were included. Within children with overweight at age 5‐6 years (N = 487, prevalence boys: 11.6%, girls: 14.6%), BMI growth patterns from birth onwards (12.8 serial measurements; SD = 3.1) were compared between children from European (69.4%) and non‐European mothers (30.6%), and between children from low (20.8%), mid (37.0%) or high SES (42.2%), based on maternal educational level.

**Results:**

BMI growth to overweight did not differ between children of European or non‐European mothers, but it did differ according to maternal SES. Children with overweight in the low and mid SES group had a lower BMI in the first 2 years of life, an earlier adiposity rebound and increased in BMI more rapidly after age 2, resulting in a higher BMI at age 7 years compared to children with overweight in the high SES group [∆BMI (kg/m^2^) between high and low SES: boys 1.43(95%CI:0.16;3.01) and girls 1.91(0.55;3.27)].

**Conclusion:**

Children with overweight from low SES have an early adiposity rebound and accelerated growth to a higher BMI at age 5‐6 years compared to children with overweight from the high SES group. These results imply that timing of critical periods for overweight development is earlier in children with a low socioeconomic background as compared to other children.

## 1 INTRODUCTION

Over the past decades there has been a worldwide increase in the prevalence of overweight and obesity among children.^1^ Children with overweight or obesity are at increased risk of adult overweight and its associated comorbidities.^2^, ^3^, ^4^, ^5^ Early prevention of childhood overweight is therefore of absolute importance and identifying critical periods in the development of childhood overweight is an essential step towards effective prevention.

BMI growth patterns differ from early age onwards between children who will develop overweight in late childhood compared to those with normal weight.^6^, ^7^, ^8^ Children with overweight have higher birthweights, and faster rates of growth in the first years of life.^6^, ^7^, ^8^, ^9^, ^10^, ^11^ Moreover, their BMI peak tends to be higher and their adiposity rebound—a renewed rise in BMI after the decrease in BMI that followed the BMI peak—tends to be earlier with a steeper increase in BMI compared to those of normal weight.^6^, ^7^, ^12^, ^13^, ^14^

This general pattern to overweight is likely to reveal differences when comparing lower vs higher socioeconomic groups, or ethnic minority populations vs the host populations. Although this has not been studied directly, previous studies provide indications to suggest such differences in growth patterns. For instance, children of low socioeconomic background are born smaller, and are more likely to be overweight at age 5 years,^15^, ^16^ showing an increased growth in the first years compared to children of high socioeconomic background. In addition, children in ethnic minority populations are more often small at birth, but have more obesity in childhood compared to children from Dutch origin.^17^, ^18^, ^19^, ^20^ This shows that they grow faster after birth, compared to children of Dutch origin. There are indications that these differences in early postnatal growth are partly due to ethnic and socioeconomic differences in feeding practises.^21^, ^22^, ^23^, ^24^, ^25^, ^26^, ^27^

Therefore, this study will investigate BMI growth patterns to overweight at age 5‐6 years using longitudinal measurements of BMI from birth onwards stratified for maternal country of origin and maternal socioeconomic background. Country of origin will be indicated by country of birth (European origin vs non‐European origin) and socioeconomic background by maternal educational level. Growth curves will be described for boys and girls separately, as growth patterns of boys and girls have been shown to differ.^28^, ^29^ Disparities in timing of the critical periods in BMI growth to overweight could help to better identify children in minority groups at risk for childhood overweight at an early stage of development.

## 2 METHODS

### 2.1 Study population

**FIGURE 1:**
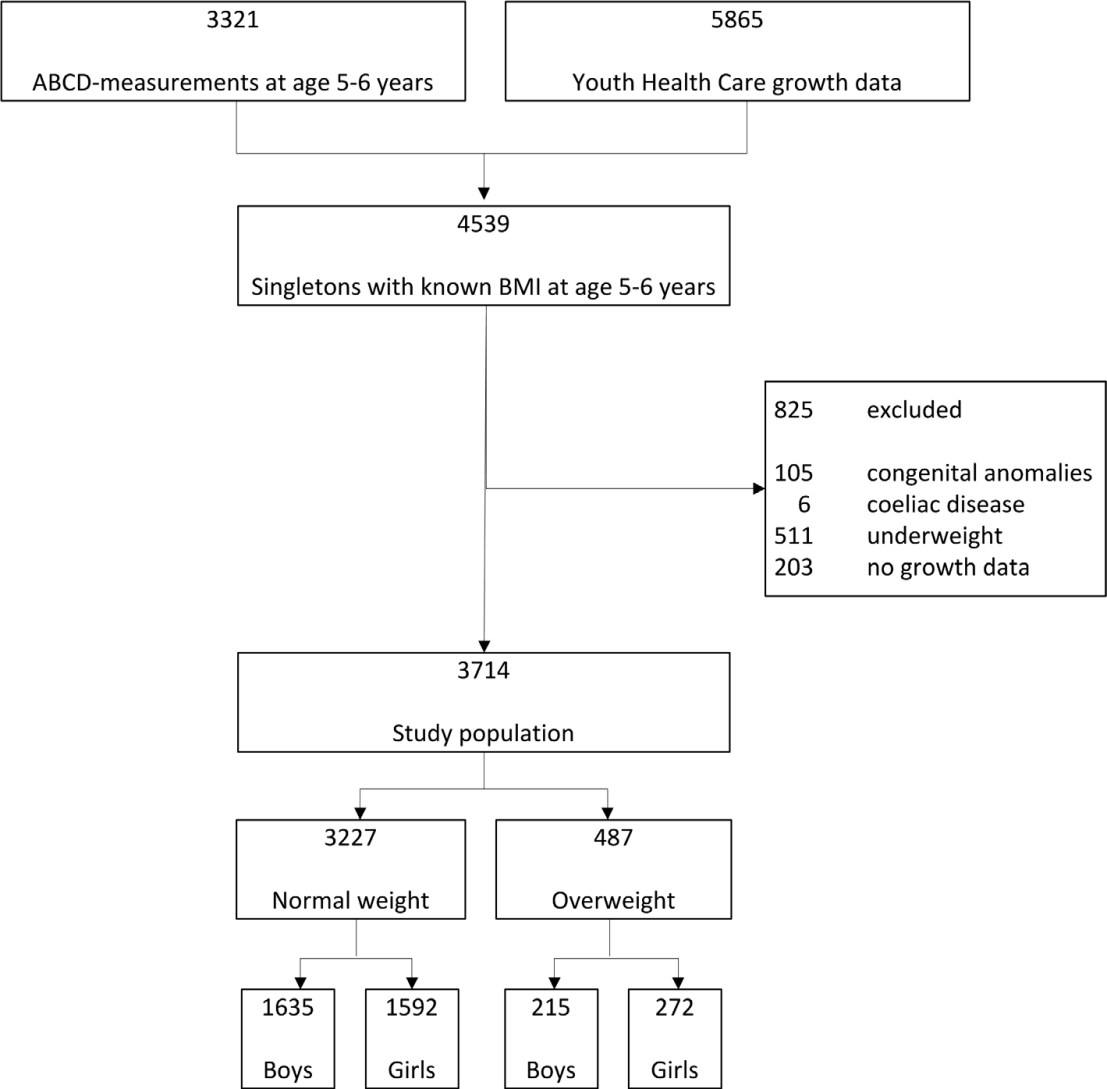
Flowchart of the study population

This study was part of the Amsterdam Born Children and their Development (ABCD) study. The aim of the prospective cohort ABCD study was to examine the association between maternal lifestyle, medical, psychosocial and environmental conditions during pregnancy and children's health at birth as well as in later life.^30^ All pregnant women in Amsterdam (January 2003‐March 2004) were approached at their first antenatal visit to an obstetric caregiver [median 13 weeks (IQR = 14‐16)] to participate. Of the 12 373 women approached, 8266 filled out the pregnancy questionnaire. Of the mothers who gave birth to a live‐born singleton, 6734 gave permission for follow‐up. In the following years, the follow‐up of growth of 5865 children was collected from files of the Youth Health Care registration of the Public Health Service in Amsterdam. Moreover, at age 5‐6 years, 3321 of the children participated in the ABCD health check, where weight and height were measured. Of the 4539 with known BMI at mean age 5.7 (SD = 0.4) years, children born with congenital anomalies (n = 105), celiac disease (n = 6), underweight (n = 511) or with missing growth data (n = 203) were excluded, leaving 3714 children in the final study sample (Figure 1). For this study, ethical approval was obtained according to the guidelines of the Declaration of Helsinki, and all procedures involving human subjects were approved by the review boards of all Amsterdam hospitals and the Registration Committee of Amsterdam. Written informed consent was obtained from all subjects.

### 2.2 BMI growth pattern

Growth data were obtained from the Youth Health Care registration of the Public Health Service in Amsterdam. Following a standard procedure, trained nurses measured at regular follow‐up moments between birth and age 6 years weight and height and BMI was calculated (mean = 12.8; SD = 3.1). For each child an individual BMI growth curve was modelled.^31^ Since the length of the child was first measured at 4 weeks of age, the BMI score at birth was extrapolated.

### 2.3 Overweight at age 5‐6 years

BMI was based on height and weight measures at 5‐6 years old, obtained from the ABCD health check (69.4%) complemented by growth measurements of the Youth Health Care registration (30.6%). Height was measured to the nearest millimetre using a Leicester portable height measure (Seca), and weight to the nearest 100 g using a Marsden weighing scale (model MS‐4102).^32^ The agreement between the two registration for height and weight could be estimated in 1531 children who were measured during the ABCD health check as well as by YHC professionals within 1 year. The intra class correlation (ICC) of height was 0.83 and for weight 0.90 which indicate good to excellent agreement. Children were divided in normal weight and overweight/obese, by sex‐ and age specific BMI cut‐off points defined by the International Obesity Task Force.^33^

### 2.4 Country of origin

Country of origin of the child was based on the country of birth of the mother and her mother, reported in the pregnancy questionnaire, to include first and second generation.^17^ The children were divided in two groups: European origin (European countries, United States, Canada, Australia and New Zealand) and other.

### 2.5 Socioeconomic status

SES was based on maternal education in years after primary school, reported in the pregnancy questionnaire and divided in three categories: low (0‐5 years), middle (6‐10 years) and high (>10 years).

### 2.6 Characteristics of the study population

The following variables were used for demographic information: maternal age (years) and maternal smoking during pregnancy (yes/no) which were self‐reported in the pregnancy questionnaire, sex (boy/girl), gestational age (days), parity (nulliparous/multiparous), and birth weight of the child (gram) were retrieved from the Youth Health Care registration.

### 2.7 Analyses

Characteristics of the study sample were described according to weight status at age 5‐6 years for boys and girls separately. Demographic differences between children with normal weight and children with overweight as well as differences between the response and non‐response group (live‐born singletons with permission to follow‐up meeting the inclusion criteria, but without growth data and/or BMI status at 5 years of age) were tested with chi‐square tests for categorical variables and two‐sample *t* tests for continuous variables.

To model the overall longitudinal BMI growth patterns of normal weight and overweight, two separate sex‐specific linear mixed models were fitted to the BMI growth data.^34^ To capture the non‐linearity of the BMI growth patterns, the fixed effect describing the relation between BMI and age were modelled with a natural spline function. The knot placement of the natural splines was based on quantiles of age. For both models, the best number of knots was chosen based on the Bayesian Information Criterion (BIC). To quantify the difference between normal weight and overweight, interaction terms were added to the model. Finally, we used a random intercept and slope to capture the correlation between BMI growth measurements from the same child.

Firstly (Part 1), differences in BMI growth patterns (BMI peak, BMI adiposity rebound) between children with normal weight and overweight were described by visual inspection. Differences between BMI at certain ages (0, 3, 6, 9 months, 1, 2, 3, 4, 5, 6, 7 years) were checked by investigating the amount of overlap of the 95% confidence intervals. If the 95% confidence intervals are not overlapping, there is some evidence that the BMI growth patterns are different.

Secondly (Part 2), the population was restricted to only children with overweight (N = 487). The same modelling procedure was followed as in Part 1. The BMI growth curves were compared for children who had overweight at age 5‐6 years of European origin and of other origin. Thirdly, to adjust for the interrelation of SES and country of origin, the population was further restricted to children with overweight from only European origin (N = 206). BMI growth curves were compared of children with overweight from high SES, middle SES and low SES groups. We did not correct for any covariates as potentially important variables as maternal smoking, birth weight and maternal weight status are strong determinants of postnatal growth, both influenced by ethnicity^17^ and SES^15^, ^16^ and are therefore part of the causal pathway. A secondary analysis with subdivision of the non‐European group into children from Turkish, Moroccan and Surinamese decent was performed to have some indication whether BMI growth curves differed between these groups. Descriptive analyses were performed in SPSS version 23 and BMI growth patterns were analyzed with R version 3.3.3.

## 3 RESULTS

### 3.1 Study population

In our study population 13.1% of the children were overweight. Boys and girls with overweight had respectively a 3.4 and 3.6 kg/m^2^ higher BMI than children with normal weight at age 5‐6 years. Overweight was more prevalent in girls (14.6%) than in boys (11.6%) and in children with a non‐European origin (24.8%) and low SES (15.6%) background (Table 1). Children with overweight more often had mothers who were younger, more often overweight themselves, multiparous and smoked more often than the mothers of children with normal weight (Table 2).

**TABLE 1:** Prevalence of overweight in our study population

		Total study population (N = 3714) (%)	Boys (N = 1850) (%)	Girls (1864) (%)
Total study population		13.1	11.6	14.6
Country of origin	European origin	8.0	7.0	8.9
	Other	24.8	21.7	27.9
Socioeconomic status (only European origin)	High SES	6.3	4.9	7.6
	Middle SES	8.9	8.3	9.4
	Low SES	15.9	16.1	15.7

Abbreviation: SES, socioeconomic status.

**TABLE 2:** Characteristics of the study population (N = 3714)

			Boys (N = 1850)		Girls (N = 1864)	
			Normal weight N = 1635 (88.4%)	Overweight N = 215 (11.6%)	Normal weight N = 1592 (85.4%)	Overweight N = 272 (14.6%)
Maternal age		Mean (SD)	31.4 (5.1)	29.3 (5.7)*	31.5 (4.9)	29.5 (5.9)*
Maternal country of origin	European origin	N (%)	1181 (72.2)	89 (41.4)*	1192 (74.9)	117 (43.0)*
	Other	N (%)	454 (27.8)	126 (58.6)	400 (25.1)	155 (57.0)
Maternal socioeconomic status	Low	N (%)	320 (19.7)	82 (38.7)*	264 (16.7)	99 (37.2)*
	Middle	N (%)	575 (35.4)	87 (41.0)	602 (38.1)	101 (38.0)
	High	N (%)	731 (45.0)	43 (20.3)	716 (45.3)	66 (24.8)
Maternal weight status	Not overweight	N (%)	1177 (78.0)	106 (54.6)*	1182 (79.3)	123 (51.3)*
	Overweight	N (%)	332 (22.0)	88 (45.4)	309 (20.7)	117 (48.7)
Maternal parity	Nulliparous	N (%)	878 (53.7)	106 (49.3)	869 (54.6)	138 (50.7)
	Multiparous	N (%)	757 (46.3)	109 (50.7)	723 (45.4)	134 (49.3)
Maternal smoking during pregnancy	Yes	N (%)	158 (9.7)	30 (14.0)*	149 (9.4)	46 (16.9)*
	No	N (%)	1476 (90.3)	185 (86.0)	1443 (90.6)	226(83.1)
Gestational age at birth (days)		Mean (SD)	279 (12)	278 (13)	279 (11)	279 (11)
Birth weight (grams)		Mean (SD)	3548 (556)	3630 (581)*	3412 (515)	3443 (542)
BMI at age 5‐6 years		Mean (SD)	15.5 (0.8)	18.9 (1.6)*	15.4 (0.9)	19.0 (1.7)*
Duration of exclusive breastfeeding^a^	None	N (%)	276 (17.7)	41 (20.3)*	250 (16.4)	57 (21.5)*
	<1 months	N (%)	125 (8.0)	26 (12.9)	124 (8.1)	32 (12.1)
	1–3 months	N (%)	459 (29.4)	69 (34.2)	434 (28.5)	67 (25.3)
	>3 months	N (%)	699 (44.8)	66 (32.7)	714 (46.9)	109 (41.1)
Sleep (h)^a^		Mean (SD)	10.5 (0.9)	10.3 (1.1)*	10. 5 (0.9)	10.3 (1.1)*
Screentime (h)^a^		Mean (SD)	1.5 (1.1)	1.9 (1.2)*	1.3 (0.9)	1.8 (1.2)*
Member of sports club^a^		N (%)	646 (53.6)	66 (56.9)	506 (44.0)	91 (58.3)*

* *P* < 0.05.

a Measured in a subset of N = 3548 (breastfeeding) and N = 1309 (sleep, screen time, membership of sports club).

### 3.2 Part 1: Total population

**FIGURE 2:**
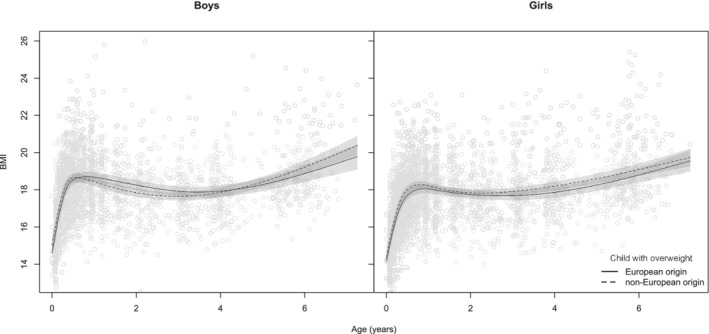
Growth patterns to normal weight and overweight at age 5‐6 years. Numbers in the different groups are: boys with normal weight (N = 1635), overweight (N = 215), girls with normal weight (N = 1592) and overweight (N = 272)

Based on the BIC, the optimal number of degrees of freedom for the natural spline function describing the relation between BMI and age was 7 for both boys and girls. Overweight boys at age 5‐6 years had a higher birth weight (Table 2) and higher BMI at birth (Table 3) compared to normal weight boys.). Figure 2 presents the average BMI growth curves of children with normal weight and overweight at age 5‐6 years. Visual inspection of this figure shows that in children with overweight, the BMI peak was higher (±1 kg/m^2^) than for children with normal weight. Although timing of the BMI peak was earlier in overweight boys than in normal weight boys, no differences were seen in girls. On the other hand, timing of the adiposity rebound was earlier in children with overweight (at age 3 years) than in children with normal weight (at age 5 years). The magnitude of the drop in BMI after the BMI peak differed between boys with overweight (ΔBMI = ±1 kg/m^2^) compared to boys with normal weight (ΔBMI = ±2 kg/m^2^), similar findings were found for girls (ΔBMI = ±0.5 kg/m^2^ for girls with overweight and ΔBMI = ±1.5 kg/m^2^ for girls with normal weight.

**TABLE 3:** ΔBMI scores (95% confidence intervals) compared with reference group

	Overweight^a^		Country of origin^b^		Socioeconomic status^c^			
	Boys	Girls	Boys	Girls	Boys		Girls	
					Low	Mid	Low	Mid
0 months	**0.45 (0.11; 0.79)**	**0.28 (0.01; 0.56)**	0.43 (−0.13;0.99)	0.24 (−0.22;0.70)	**−1.41 (−2.36;‐0.45)**	−0.80 (−1.61;0.01)	−0.23 (−1.12;0.65)	−0.23 (−0.9;0.43)
3 months	**1.02 (0.84; 1.20)**	**0.83 (0.68; 0.98)**	0.34 (−0.03;0.72)	0.32 (−0.01;0.65)	**−1.15 (−1.87;‐0.43)**	−0.58 (−1.18;0.02)	0.29 (−0.41;1.00)	−0.21 (−0.73;0.32)
6 months	**1.09 (0.92; 1.26)**	**0.95 (0.80; 1.09)**	0.11 (−0.24;0.47)	0.31 (−0.00;0.62)	**−0.99 (−1.72;‐0.26)**	−0.47 (−1.07;0.13)	0.28 (−0.41;0.97)	−0.28 (−0.78;0.23)
9 months	**1.03 (0.86; 1.20)**	**1.08 (0.93; 1.23)**	−0.11 (−0.47;0.25)	0.25 (−0.07;0.56)	**−0.93 (−1.62;‐0.23)**	−0.47 (−1.04;0.10)	−0.13 (−0.78;0.52)	−0.40 (−0.88;0.08)
1 year	**1.01 (0.85; 1.18)**	**1.20 (1.06; 1.34)**	−0.25 (−0.59;0.10)	0.17 (−0.13;0.47)	**−0.88 (−1.59;‐0.17)**	−0.49 (−1.08;0.09)	−0.45 (−1.10;0.21)	−0.48 (−0.96;0.01)
2 years	**1.29 (1.13; 1.45)**	**1.47 (1.32; 1.61)**	−0.41 (−0.77;‐0.05)	0.08 (−0.23;0.40)	−0.69 (−1.40;0.01)	−0.59 (−1.18;‐0.01)	−0.31 (−0.92;0.30)	−0.28 (−0.73;0.17)
3 years	**1.63 (1.48; 1.78)**	**1.95 (1.81; 2.09)**	−0.27 (−0.62;0.08)	0.23 (−0.09;0.54)	−0.42 (−1.20;0.36)	−0.52 (−1.16;0.13)	0.38 (−0.27;1.03)	0.10 (−0.38;0.58)
4 years	**2.06 (1.89; 2.22)**	**2.53 (2.38; 2.68)**	−0.06 (−0.47;0.35)	0.34 (−0.03;0.71)	−0.05 (−0.91;0.81)	−0.22 (−0.93;0.50)	**0.91 (0.18;1.65)**	0.42 (−0.13;0.96)
5 years	**2.66 (2.48; 2.83)**	**3.04 (2.88; 3.20)**	0.14 (−0.31;0.60)	0.35 (−0.07;0.76)	0.40 (−0.56;1.36)	0.25 (−0.56;1.06)	**1.32 (0.47;2.17)**	**0.69 (0.06;1.32)**
6 years	**3.39 (3.18; 3.59)**	**3.48 (3.29; 3.67)**	0.35 (−0.22;0.92)	0.29 (−0.23;0.81)	0.90 (−0.30;2.10)	0.83 (−0.19;1.85)	**1.64 (0.59;2.70)**	**0.92 (0.13;1.71)**
7 years	**4.19 (3.86; 4.52)**	**3.89 (3.58; 4.19)**	0.55 (−0.25; 1.35)	0.20 (−0.51; 0.91)	**1.43 (0.16; 3.01)**	**1.48 (0.12; 2.84)**	**1.91 (0.55; 3.27)**	**1.13 (0.1; 2.16)**

*Note:* Bold numbers indicate no overlapping 95% confidence intervals.

a ΔBMI score between children with normal weight [reference, boys (N = 1635), girls (N = 1592)] and children with overweight [boys (N = 215), girls (N = 272)] at age 5‐6 years.

b ΔBMI score between children from European origin with overweight [reference, boys (N = 89), girls (N = 117)] and children from non‐European origin with overweight [(boys, N = 126), girls (N = 155)].

c Only children from European origin included (N = 206). ΔBMI score between children from high socioeconomic status with overweight [reference, boys (N = 35), girls (N = 55)] and middle SES with overweight [(boys (N = 36), girls (N = 45)]/low SES with overweight [(boys (N = 18), girls (N = 17)].

### 3.3 Part 2: Children with overweight at age 5‐6 years

#### 3.3.1 BMI growth curves in children with overweight at age 5‐6 years: European vs non‐European origin children

**FIGURE 3:**
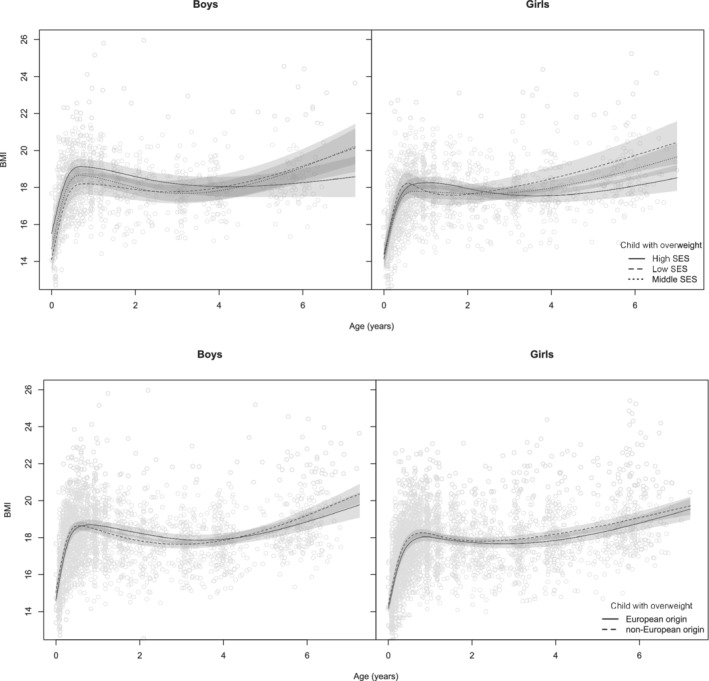
Growth patterns to overweight at age 5‐6 years by maternal country of origin (lower panel) and socioeconomic status (only women from European origin; upper panel). Numbers in the different groups are: boys from low SES (N = 82), mid SES (N = 87), high SES (N = 43), girls from low SES (N = 99), mid SES (N = 101), high SES (N = 66). Boys from European origin (N = 89), non‐European origin (N = 126), girls from European origin (N = 117) and non‐European origin (N = 155). SES, socioeconomic status

Based on the BIC, the optimal number of degrees of freedom for the natural spline function describing the relation between BMI and age was 5 for both boys and girls. Figure 3 (lower panel) presents the BMI growth curves to overweight of boys and girls from European origin and non‐European origin. No differences were found in birth weight and subsequent growth patterns to overweight between both populations (Table 3 and Table S3). The secondary analysis indicated differences within the non‐European group, which were most pronounced for children from Turkish decent. Girls with overweight at age 5‐6 from Turkish decent showed the highest BMI peak, the earliest adiposity rebound and the highest BMI from age 2 onwards (Figure SS1).

#### 3.3.2 BMI growth curves in children with overweight at age 5‐6 years: children from lower vs higher SES background

Based on the BIC, the optimal number of degrees of freedom for the natural spline function describing the relation between BMI and age was 4 for both boys and girls. Figure 3 (upper panel) presents the growth to overweight for children from different SES groups, in children of European origin only. No differences in the growth patterns to overweight were found between children of low and middle SES, but both groups differed from the growth patterns observed in the higher SES group. Although timing and magnitude of the BMI peak was similar for all SES groups, the low/middle SES groups had a lower BMI during the first 2 years (only statistically significant for the low group; Table 3) and an earlier adiposity rebound (Figure 3) than children from the high SES group. After age 3 years BMI increased rapidly in the low/middle SES group and at age 7 years the children with overweight in low/middle SES groups had a higher mean BMI than those in the high SES group (Table 3).

## 4 DISCUSSION

This study investigated socioeconomic and country of origin inequalities in BMI growth patterns to overweight at age 5‐6 years, for boys and girls separately. No differences in BMI growth patterns to overweight were found when comparing European origin children with non‐European origin children. However, children with overweight in the low/middle SES group had a lower BMI during the first 2 years, an earlier adiposity rebound and a higher BMI at age 5‐6 years compared to children with overweight in the high SES group.

### 4.1 Strengths and Limitations

An important strength of this study was its longitudinal design with, on average, 13 standardized measurements at standard times performed by trained health care professionals in municipal Youth Health Care centers. However, this study had some limitations as well. First, we cannot rule out that there are no disparities in growth patterns to overweight in individual country of origin groups. Earlier research within the ABCD study showed that from 0 to3 years, Surinamese children have lower BMIs and Moroccan and Turkish children have higher BMIs compared to the Dutch children,^29^ while all three groups have higher percentages of overweight at age 5‐6 years,^35^, ^36^ indicating that growth patterns to overweight might differ between specific non‐European origin groups. Our secondary analysis with more subdivisions of the non‐European group into Turkish, Moroccan and Surinamese children confirmed these differences in growth patterns in children with overweight, with children from Turkish decent (especially girls) showing the most detrimental BMI growth pattern to overweight. However, we should keep in mind that the individual groups were not large and therefore were combined into one non‐European origin group in our main analysis. More research is needed to confirm our preliminary findings with larger numbers. Second, we might have underestimated the inequalities in BMI growth patterns, due to the lower prevalence of overweight in this study (girls: 14.6%; boys: 11.6%) compared to the prevalence nationwide (girls: 18.5%; boys: 14.6%).^37^ Fewer children with overweight, results in lower power to generate differences in growth patterns.

### 4.2 Comparison with the literature

This study confirms earlier reported growth patterns to overweight in the total population: children with overweight had higher birth weight, a higher BMI peak and an earlier adiposity rebound.^6^, ^7^, ^12^, ^13^, ^14^ Moreover, to our knowledge, this is the first study that compared longitudinal growth patterns to childhood overweight in separate groups with a higher risk for overweight and focusses on socioeconomic and country of origin inequalities in BMI growth patterns for boys and girls separately. This study found a higher prevalence of overweight in the non‐European origin children at age 5‐6 years, but a similar growth pattern to overweight for children of European origin and non‐European origin. Therefore, this study adds to the literature that ethnic inequalities in growth patterns, for instance, accelerated growth in the first year, might simply be a reflection of the higher prevalence of overweight in these groups,^29^, ^38^, ^39^, ^40^ rather than a specific ethnic growth pattern. Regarding SES, this study also found a higher prevalence of overweight in the low SES group compared to the high SES group. In contrast to the country of origin groups, however, also the growth patterns to overweight were different for the low and high SES group. The BMI of children with overweight at age 5‐6 was lower in the low SES group during the first two years, but higher at age 5‐6 years compared to the high SES group. Others did not distinguish between children with and without overweight, but did find similar patterns when socioeconomic inequalities in BMI growth were studied. In Generation R and ALSPAC, children in the low SES group had a lower BMI at age 2 years,^41^, ^42^ but a higher BMI at age 6 and 10 years.^41^, ^43^ This suggests that, socioeconomic inequalities in growth patterns do not simply result from socioeconomic inequalities in prevalence of overweight, but that children with a low socioeconomic background who end up being overweight, truly have a different growth pattern towards that overweight than children of a high socioeconomic background.

### 4.3 Underlying mechanisms

There are different mechanisms that could underlie the SES differences found in our cohort, but infant feeding patterns can probably be ruled out. Although children with low SES in our cohort had shorter breastfeeding duration and earlier introduction of solid foods,^44^ which is associated with the development of childhood overweight,^45^, ^46^ they had lower BMIs in the first 2 years.

Moreover, within our cohort, differences in feeding style did not mediate the association of low SES with weight‐for‐length gain from 1‐5 years.^44^ The lower BMI in the first 2 years might be explained by maternal smoking. Maternal smoking during pregnancy impairs foetal growth and causes low birth weight,^47^, ^48^, ^49^ which often leads to catch‐up growth and an increased risk of overweight in childhood.^50^ In this study, 35% of the mothers with a low SES smoked during pregnancy, this was even higher (51%) if their children were overweight at age 5‐6 years (Table S5). A previous study within our cohort showed that maternal smoking during pregnancy largely attributed to the higher prevalence of infants born small‐for‐gestational age in the low SES group.^51^ Therefore, the socioeconomic differences in growth patterns to overweight observed in infancy could be explained by maternal smoking during pregnancy. Furthermore, in the earlier mentioned study on the association between SES and weight‐for‐length gain from 1 to 5 years, the most important mediator was maternal pre‐pregnancy BMI.^44^ The increased growth after two years might therefore be a consequence of maternal overweight, as prevalence of maternal overweight is higher in women of low SES.^52^, ^53^, ^54^, ^55^ related to an unhealthier lifestyle. Important childhood mediators in the inverse association between SES and childhood overweight at age 5 years are maternal TV watching, consuming breakfast and TV watching by the child.^56^ More in general, not participating in organized sport was found to be associated with increased BMI *z*‐scores,^57^ and children with overweight from a low socioeconomic background sleep shorter than their peers with normal weight.^58^, ^59^ In our study, children in the low SES group had more often mothers with overweight, they watched more TV and slept less, and were less often a member of a sports club (Table S5). Another explanation for the accelerated growth of children from the low SES group might be that mothers/parents perceive the child's overweight as normal and therefore take no action to change their lifestyle. Maternal underestimation of child's weight is more common in mother from low SES compared to mother from high SES.^60^ The recognition of child's overweight is an important first step for the success of interventions aimed at prevention of further accelerated weight gain.

### 4.4 Implications

This study showed that children with overweight from the low/middle SES group have lower BMIs in the first year compared to the high SES group, but after 2 years, these groups accelerated in growth and had a higher BMI at age 5‐6 years. Therefore, it is important to closely monitor children with a low SES background as their weight might be in the normal range before the age of 2, but this does not prevent them from being at risk of developing overweight later on. An early adiposity rebound should be an indicator for extra care, but to identify the adiposity rebound, it is necessary to measure anthropometrics frequently, for instance every 3‐4 months, in both infancy and early childhood. This is of foremost importance as BMI in childhood is a predictor of adult BMI^3^, ^4^, ^5^ and childhood SES has a greater influence on adult BMI and the prevalence of overweight/obesity than adult SES.^61^ A combination of both a low SES and high BMI in early childhood might therefore be most detrimental for adult BMI.

## 5 CONCLUSION

To our knowledge, this is the first study that compares growth patterns to overweight between children from different socioeconomic and country of origin groups. Stratification by SES and country of origin background showed that the development of overweight is broadly similar for children from European origin and non‐European origin. However, children with overweight from a low SES background had a lower BMI during the first 2 years, but an earlier adiposity rebound and a more rapid BMI development after 2 years compared to children in the high SES group. Our results imply that within the Youth Health Care a distinction should be made according to children's SES in determining the critical periods in childhood growth.

## CONFLICT OF INTEREST STATEMENT

No conflict of interest was declared.

## AUTHOR CONTRIBUTIONS

A. J. J. M. O and M. H. P. H. conducted the analysis. A. J. J. M. O drafted the initial manuscript. All authors were involved in setting up the study, writing the paper and had final approval of the submitted and published version.

## Supporting information


**Figure S1**.
**Table S1**. Non‐response analysis.
**Table S2**. BMI (kg/m^2^) of boys and girls with normal weight and overweight at age 5‐6 years.
**Table S3**. BMI (kg/m^2^) of overweight boys and girls at age 5‐6 years, split on maternal country of origin.
**Table S4**. BMI (kg/m^2^) of overweight boys and girls at age 5‐6 years, split on maternal socioeconomic status (only children from European origin Included).
**Table S5**. SES differences in obesogenic environment (only children from European origin included, N = 2579).
