# Supplementary material for: Growth patterns from birth to overweight at age 5‐6 years of children with various backgrounds in socioeconomic status and country of origin: the ABCD study
Source: Pediatr Obes. 2020 Apr 1;15(8):e12635. doi: 10.1111/ijpo.12635 (PMC7507194; doi:10.1111/ijpo.12635)
Supplement: Supplementary file 1 — Figure S1. Table S1. Non‐response analysis. Table S2. BMI (kg/m2) of boys and girls with normal weight and overweight at age 5‐6 years. Table S3. BMI (kg/m2) of overweight boys and girls at age 5‐6 years, split on maternal country of origin. Table S4. BMI (kg/m2) of overweight boys and girls at age 5‐6 years, split on maternal socioeconomic status (only children from European origin Included). Table S5. SES differences in obesogenic environment (only children from European origin included, N = 2579). [file IJPO-15-e12635-s001.doc]

Supplementary figure 1


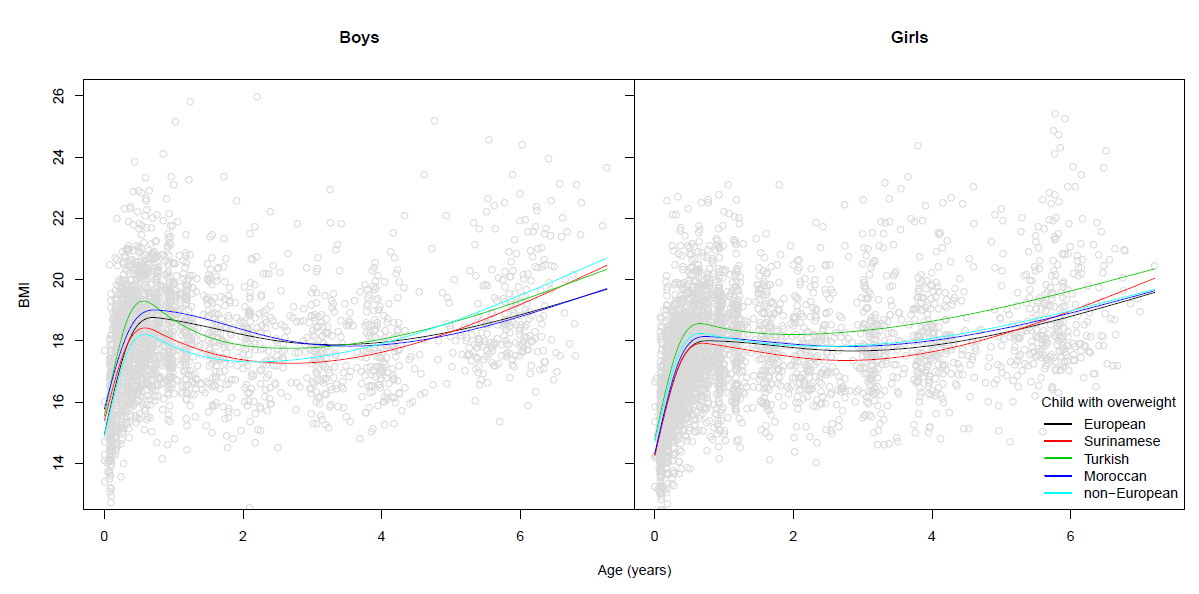


Supplementary table 1: Non-response analysis.

|  |  | **N** | **Response**  **(N=3714)** | **N** | **Non-response#**  **(N=2077)** | **P-value** |
| --- | --- | --- | --- | --- | --- | --- |
| **Maternal age** | *Mean (SD)* | 3714 | 31.2 (5.1) | 2430 | 30.4 (5.1) | P<0.001 |
| **Ethnicity (%)** | European origin | 2579 | 69.4 | 1678 | 68.9 | P=0.672 |
|  | Non-European origin | 1135 | 30.6 | 755 | 31.1 |  |
| **Socioeconomic status (%)** | Low | 765 | 20.8 | 531 | 22.1 | P=0.055 |
|  | Middle | 1365 | 37.0 | 935 | 38.8 |  |
|  | High | 1556 | 42.2 | 942 | 39.1 |  |
| **Maternal weight status (%)** | Normal weight | 2588 | 75.4 | 1747 | 78.8 | P=0.009 |
|  | Overweight | 846 | 24.6 | 469 | 21.2 |  |
| **Parity (%)** | Nulliparous | 1991 | 53.6 | 1431 | 58.9 | P<0.001 |
|  | Multiparous | 1723 | 46.4 | 999 | 41.1 |  |
| **Maternal smoking during pregnancy (%)** | Yes | 383 | 10.3 | 199 | 8.2 | P=0.006 |
|  | No | 3330 | 89.7 | 2226 | 91.8 |  |
| **Gestational age (weeks)** | *Mean (SD)* | 3692 | 279 (12) | 2398 | 278 (15) | P<0.001 |
| **BMI at age 5-6 years** | *Mean (SD)* | 3714 | 15.9 (1.5) | 128 | 15.8 (1.5) | P=0.347 |
| **Birth weight (grams)** | *Mean (SD)* | 3702 | 3487 (544) | 2409 | 3418 (589) | P<0.001 |
| **Sex (%)** | Boy | 1850 | 49.8 | 1212 | 49.9 | P=0.938 |
|  | Girl | 1864 | 50.2 | 1216 | 50.1 |  |

# Non-response sample are live-born singletons with permission to follow-up, meeting the inclusion criteria, but without growth data available and/or BMI status at 5 years of age.

**Supplementary Table 2 – BMI (kg/m^2^) of boys and girls with normal weight and overweight at age 5-6 years**

|  | **Boys** | | | | **Girls** | | | |
| --- | --- | --- | --- | --- | --- | --- | --- | --- |
|  | **Normal weight**  **N=1635** | | **Overweight**  **N=215** | | **Normal weight**  **N=1592** | | **Overweight**  **N=272** | |
|  | BMI | (95% CI) | BMI | (95% CI) | BMI | (95% CI) | BMI | (95% CI) |
| 0 months | 13.56 (13.45;13.67)  16.79 (16.73;16.85)  17.34 (17.29;17.4)  17.69 (17.63;17.75)  17.56 (17.5;17.61)  16.7 (16.65;16.76)  16.12 (16.07;16.18)  15.82 (15.76;15.88)  15.71 (15.65;15.77)  15.73 (15.65;15.81)  15.82 (15.69;15.95) | | 14.02 (13.69;14.34)  17.81 (17.64;17.98)  18.44 (18.27;18.6)  18.72 (18.56;18.88)  18.57 (18.41;18.72)  17.99 (17.84;18.15)  17.75 (17.61;17.9)  17.88 (17.72;18.03)  18.36 (18.2;18.53)  19.11 (18.93;19.3)  20.01 (19.71;20.31) | | 13.48 (13.38; 13.59)  16.01 (15.96; 16.07)  16.81 (16.76; 16.87)  17.13 (17.07; 17.19)  16.96 (16.9; 17.01)  16.3 (16.25; 16.36)  15.83 (15.78; 15.88)  15.54 (15.48; 15.6)  15.44 (15.38; 15.51)  15.48 (15.41; 15.56)  15.60 (15.47; 15.72) | | 13.76 (13.5; 14.02)  16.84 (16.7; 16.99)  17.76 (17.62; 17.89)  18.21 (18.07; 18.35)  18.16 (18.03; 18.29)  17.77 (17.64; 17.9)  17.78 (17.66; 17.91)  18.08 (17.94; 18.21)  18.49 (18.34; 18.63)  18.96 (18.79; 19.14)  19.48 (19.21; 19.76) | |
| 3 months |
| 6 months |
| 9 months |
| 1 year |
| 2 years |
| 3 years |
| 4 years |
| 5 years |
| 6 years |
| 7 years |

**Supplementary Table 3 – BMI (kg/m^2^) of overweight boys and girls at age 5-6 years, split on maternal country of origin**

|  | **Boys** | | | | **Girls** | | | |
| --- | --- | --- | --- | --- | --- | --- | --- | --- |
|  | **European origin**  **N=89** | | **Non-European origin**  **N=126** | | **European origin**  **N=117** | | **Non-European origin**  **N=155** | |
|  | BMI | (95% CI) | BMI | (95% CI) | BMI | (95% CI) | BMI | (95% CI) |
| 0 months | 14.6 (14.18;15.02)  17.43 (17.15;17.72)  18.52 (18.25;18.79)  18.69 (18.41;18.97)  18.67 (18.41;18.93)  18.24 (17.97;18.52)  17.91 (17.64;18.18)  17.92 (17.61;18.24)  18.27 (17.92;18.63)  18.87 (18.41;19.32)  19.59 (18.95;20.23) | | 15.03 (14.66;15.40)  17.78 (17.53;18.02)  18.64 (18.4;18.87)  18.58 (18.35;18.82)  18.43 (18.2;18.65)  17.84 (17.61;18.07)  17.64 (17.42;17.87)  17.86 (17.6;18.12)  18.42 (18.13;18.71)  19.21 (18.86;19.56)  20.14 (19.65;20.63) | | 14.16 (13.81;14.51)  16.56 (16.31;16.8)  17.69 (17.45;17.93)  18.01 (17.77;18.25)  18.04 (17.81;18.27)  17.75 (17.51;17.98)  17.68 (17.44;17.92)  17.86 (17.58;18.14)  18.25 (17.94;18.57)  18.79 (18.39;19.19)  19.41 (18.87;19.95) | | 14.40 (14.10;4.71)  16.88 (16.67;17.09)  18.00 (17.80;18.20)  18.26 (18.06;18.46)  18.21 (18.01;18.41)  17.83 (17.62;18.03)  17.90 (17.70;18.11)  18.19 (17.95;18.44)  18.60 (18.33;18.87)  19.08 (18.75;19.42)  19.61 (19.15;20.06) | |
| 3 months |
| 6 months |
| 9 months |
| 1 year |
| 2 years |
| 3 years |
| 4 years |
| 5 years |
| 6 years |
| 7 years |

**Supplementary Table 4 – BMI (kg/m^2^) of overweight boys and girls at age 5-6 years, split on maternal socioeconomic status (only children from European origin Included)**

|  | **Boys** | | | | | | **Girls** | | | | | |
| --- | --- | --- | --- | --- | --- | --- | --- | --- | --- | --- | --- | --- |
|  | **Low**  **N=18** | | **Mid**  **N=36** | | **High**  **N=35** | | **Low**  **N=17** | | **Mid**  **N=45** | | **High**  **N=55** | |
|  | BMI | 95% CI | BMI | 95% CI | BMI | 95% CI | BMI | 95% CI | BMI | 95% CI | BMI | 95% CI |
| 0 months | 14.11 (13.34;14.87)  16.65 (16.06;17.23)  17.97 (17.38;18.55)  18.19 (17.63;18.75)  18.18 (17.6;18.75)  17.89 (17.32;18.46)  17.77 (17.14;18.39)  17.99 (17.31;18.68)  18.48 (17.74;19.23)  19.16 (18.24;20.08)  19.93 (18.73;21.14) | | 14.71(14.13; 15.29)  17.21(16.79; 17.63)  18.48(18.06; 18.91)  18.65(18.25; 19.05)  18.56(18.15; 18.97)  17.99(17.58; 18.39)  17.67(17.23; 18.12)  17.82(17.34; 18.31)  18.33(17.79; 18.87)  19.09(18.42; 19.76)  19.99(19.10; 20.88) | | 15.51 (14.95;16.08)  17.79 (17.37;18.22)  18.96 (18.53;19.39)  19.12 (18.71;19.53)  19.06 (18.64;19.48)  18.58 (18.17;19.00)  18.19 (17.72;18.65)  18.04 (17.52;18.56)  18.08 (17.48;18.69)  18.26 (17.49;19.03)  18.51 (17.48;19.53) | | 14.17 (13.4; 14.94)  16.94 (16.34; 17.55)  18.17 (17.57; 18.76)  18.06 (17.5; 18.63)  17.81 (17.24; 18.37)  17.63 (17.1; 18.16)  17.98 (17.42; 18.55)  18.47 (17.84; 19.11)  19.07 (18.33; 19.8)  19.73 (18.82; 20.64)  20.43 (19.27; 21.59) | | 14.17 (13.68; 14.66)  16.44 (16.06; 16.83)  17.61 (17.23; 17.98)  17.79 (17.44; 18.15)  17.78 (17.42; 18.13)  17.66 (17.33; 17.99)  17.7 (17.35; 18.06)  17.98 (17.58; 18.38)  18.43 (17.97; 18.89)  19.01 (18.43; 19.58)  19.65 (18.9; 20.4) | | 14.40 (13.96; 14.85)  16.65 (16.30; 17.00)  17.88 (17.54; 18.23)  18.19 (17.87; 18.52)  18.25 (17.93; 18.58)  17.94 (17.64; 18.25)  17.60 (17.28; 17.93)  17.56 (17.19; 17.93)  17.75 (17.32; 18.18)  18.09 (17.55; 18.63)  18.52 (17.81; 19.22) | |
| 3 months |
| 6 months |
| 9 months |
| 1 year |
| 2 years |
| 3 years |
| 4 years |
| 5 years |
| 6 years |
| 7 years |

**Supplementary table 5 - SES differences in obesogenic environment (only children from European origin included, N=2579)**

|  |  |  | **Low SES** | | **Middle SES** | | **High SES (ref)** | |
| --- | --- | --- | --- | --- | --- | --- | --- | --- |
|  |  |  | Normal weight  N=172 (83%) | Overweight  N=35 (17%) | Normal weight  N=793 (91%) | Overweight  N=78 (9%) | Normal weight  N=1303 (94%) | Overweight  N=88 (6%) |
| **Maternal weight status** | Normal weight | *N (%)* | 131 (74.9)* | 14 (42.4)* | 618 (78.7)* | 46 (61.3)* | 1132 (87.1) | 63 (75.9) |
|  | Overweight | *N (%)* | 44 (25.1)* | 19 (57.6)* | 167 (21.3)* | 29 (38.7)* | 168 (12.9) | 20 (24.1) |
| **Maternal smoking during pregnancy** | Yes | *N (%)* | 59 (31.9)* | 18 (51.4)* | 110 (13.2)* | 17 (21.0)* | 62 (4.6) | 7 (7.8) |
|  | No | *N (%)* | 126 (68.1)* | 17 (48.6)* | 721 (86.8)* | 64 (79.0)* | 1288 (95.4) | 83 (92.2) |
| **Birth weight (gram)** |  | *Mean (SD)* | 3432 (570) | 3472 (411) | 3492 (559) | 3667 (631)* | 3545 (514) | 3586 (515) |
| **Duration of exclusive breastfeeding^#^** | none | *N (%)* | 48 (27.9)* | 17 (48.6)* | 160 (20.2)* | 21 (26.9)* | 184 (14.1) | 13 (14.8) |
|  | <1 months | *N (%)* | 26 (15.1)* | 6 (17.1)* | 55 (6.9)* | 14 (17.9)* | 63 (4.8) | 4 (4.5) |
|  | 1-3 months | *N (%)* | 50 (29.1)* | 7 (20.0)* | 228 (28.8)* | 20 (25.6)* | 339 (26.0) | 21 (23.9) |
|  | >3 months | *N (%)* | 48 (27.9)* | 5 (14.3)* | 350 (44.1)* | 23 (29.5)* | 717 (55.0) | 50 (56.8) |
| **Sleep (hours)^#^** |  | *Mean (SD)* | 10.3 (1.1)* | 9.5 (1.6)* | 10.7 (0.8) | 10.6 (0.9) | 10.7 (0.7) | 10.6 (0.8) |
| **Screentime (hours)^#^** |  | *Mean (SD)* | 1.6 (1.0)* | 1.9 (0.7)* | 1.4 (0.8)* | 1.5 (0.8)* | 1.1 (0.7) | 1.2 (0.6) |
| **Member of sports club^#^** | Yes | *N (%)* | 49 (43.8)* | 12 (63.2)* | 363 (56.5) | 32 (64.0)* | 668 (56.9) | 36 (54.5) |
|  | No | *N (%)* | 63 (56.2)* | 7 (36.8)* | 279 (43.5) | 18 (36.0)* | 506 (43.1) | 30 (45.5) |

* p<0.05
